# Global Research Trends and Hotspots in Gene Editing and Stem Cell Therapies for Neurodegenerative Diseases: Bibliometric and Visualization Analysis

**DOI:** 10.2196/83709

**Published:** 2026-03-09

**Authors:** Lijun Xiang, Yun Xiao, Ming Cai, Jing Qin, Ting Wang, Xueming Xiang, Jun Ke, Ganlin Peng

**Affiliations:** 1 Department of General Medicine, Zhangjiajie Hospital Affiliated to Hunan Normal University, Zhangjiajie, Hunan, 427000, China; 2 Department of Respiratory and Critical Care Medicine, Changsha Central Hospital, No. 161, Shaoshan South Road, Tianxin District, Changsha, Hunan, 410004, China, 86 18874042983; 3 Department of Neurology, Zhangjiajie Hospital Affiliated to Hunan Normal University, Zhangjiajie, Hunan, 427000, China; 4 Department of Infectious Diseases, Zhangjiajie Hospital Affiliated to Hunan Normal University, Zhangjiajie, Hunan, 427000, China

**Keywords:** neurodegenerative diseases, gene editing technology, stem cell therapy, bibliometric analysis, clinical translation

## Abstract

**Background:**

Neurodegenerative diseases are a major and growing global health burden. Their pathogenesis is complex, and effective therapies remain limited. Gene editing and stem cell–based strategies are reshaping the therapeutic landscape. However, the field has not been systematically examined through bibliometric analysis.

**Objective:**

We aimed to define the intellectual landscape of global research on gene editing and stem cell therapy for neurodegenerative diseases from 2005 to 2024, highlight evolving hotspots, track the field’s evolution, and identify major bottlenecks limiting clinical translation.

**Methods:**

We retrieved 1821 publications from the Web of Science Core Collection (2005-2024). We performed a multidimensional bibliometric analysis using CiteSpace and VOSviewer. We assessed publication output, country and institutional contributions, key authors and journals, co-cited references, and keyword networks. These analyses were used to track the field’s evolution and pinpoint emerging themes.

**Results:**

In total, 9978 researchers from 90 countries and 2515 institutions contributed to this literature. Annual publications increased from 28 in 2005 to 179 in 2024, with stepwise growth over time. The United States ranked first in output (n=780) and in citation impact (total local citation score=2784; total global citation score=40,009). China and India ranked second and fifth in output, respectively, but their average citation impact was lower than that of the leading countries. The University of California, San Francisco, and Johns Hopkins University remained consistently influential. Boulis NM, Bankiewicz KS, and Feldman EL were among the most prominent contributors. *Molecular Therapy* was the leading journal in this area. Keyword analyses pointed to a growing intersection between genetics and immunology. Major topics included nanotechnology-based delivery, adeno-associated virus vectors, small interfering RNA, intrathecal microsphere injection, autophagy, blood-brain barrier (BBB) targeting, clustered regularly interspaced short palindromic repeats (CRISPR)/CRISPR-associated protein 9 (Cas9), and induced pluripotent stem cells. Burst detection highlighted “open label” as a recent hotspot. This likely reflects rising translational activity and early clinical testing.

**Conclusions:**

The field is moving from technology development toward clinical translation. Anglo-American countries currently drive both productivity and influence. China and India contribute heavily to volume but need a stronger impact. CRISPR/induced pluripotent stem cell platforms and BBB-focused delivery remain central frontiers. The rise of “open-label” studies suggests accelerating clinical momentum. Future progress will require safer and more efficient delivery, clearer standards, and larger global consortia to harmonize protocols and speed translation.

## Introduction

Neurodegenerative diseases (NDs) comprise a group of progressive disorders characterized by neuronal loss and functional decline, leading to worsening cognitive, behavioral, and/or motor impairments over time [1]. Common NDs include Alzheimer disease (AD), Parkinson disease (PD), amyotrophic lateral sclerosis (ALS), and Huntington disease (HD). Globally, more than 50 million individuals are affected, and the prevalence is rising with population aging, creating a growing public health and economic burden [2]. Although decades of research have clarified key pathological hallmarks, such as β-amyloid deposition, tau hyperphosphorylation, and α-synuclein aggregation [3,4], most available therapies remain largely symptomatic and do not reliably halt or reverse disease progression. Only a limited number of drugs, including donepezil and levodopa, are approved for clinical use, and their benefits are modest and often constrained by adverse effects. Accordingly, there remains a strong need for therapies that modify disease mechanisms rather than symptoms alone [5,6].

Recent advances in gene editing and stem cell technologies have created new therapeutic possibilities for NDs [7,8]. The clustered regularly interspaced short palindromic repeats (CRISPR)/CRISPR-associated protein 9 (Cas9) system enables precise perturbation of disease-relevant genes and can be used to correct pathogenic variants or modulate aberrant protein expression, including targets such as *APP*, *SNCA*, *SOD1*, and *C9orf72*. In parallel, induced pluripotent stem cells (iPSCs) can be differentiated into functional neuronal populations, providing a platform for cell replacement strategies. For example, CRISPR-based *SOD1* silencing delays motor neuron degeneration in ALS models, and transplantation of iPSC-derived dopaminergic neurons has shown potential to improve motor outcomes in clinical studies of PD [9]. Notably, the convergence of these approaches is becoming an active research direction. The Cas9nVQR editing tool can efficiently modulate gene dosage in human iPSCs and has been reported to ameliorate AD-related phenotypes, including amyloid-β (Aβ) secretion and tau hyperphosphorylation [10]. Gene editing may also be applied to improve graft survival and functional engraftment by reducing immune rejection after transplantation [11]. However, key challenges remain, including off-target editing, tumorigenic risk of stem cell–derived products, and safe and efficient delivery in vivo. Addressing these barriers will be essential for clinical translation.

Bibliometric analysis applies quantitative methods to published literature to map the knowledge structure and research dynamics within a field. Using tools, such as CiteSpace, VOSviewer, and HistCite, bibliometrics can identify influential contributors and core documents, track emerging topics, and evaluate scholarly impact. It can also reveal collaboration networks and cross-disciplinary opportunities, thereby informing more strategic allocation of research efforts and resources. In addition, bibliometric evidence can help policymakers understand field trajectories and support evidence-based decision-making [12-14]. In NDs, prior bibliometric studies have focused on selected diagnostic and therapeutic topics [15,16]. However, gene editing and stem cell therapy represent rapidly expanding and increasingly intersecting frontiers, and a systematic bibliometric assessment of their progress, technical barriers, and translational trajectories in NDs remains lacking. Therefore, we performed a bibliometric and visual analysis of global research on gene editing and stem cell therapy for NDs from 2005 to 2024. By integrating publication trends, institutional collaboration networks, co-citation clustering, and keyword burst detection, this study aims to define the field’s intellectual landscape, highlight evolving hotspots, and identify major bottlenecks limiting clinical translation [17].

## Methods

### Data Sources and Search Strategies

This study systematically retrieved articles published between January 2005 and December 2024 based on the Web of Science Core Collection (WoSCC) database. The retrieval strategy revolved around 2 major themes: NDs (including AD, PD, HD, etc) and gene editing and stem cell therapy technologies (covering CRISPR/Cas9, base editing, stem cell transplantation, etc). The 2 groups of terms were connected by the Boolean operator “AND.” For the ND group, an extended search formula was used as follows: TS=(“Neurodegenerative Diseases” OR “Degenerative Neurologic Disorder” OR “Nervous System Degenerative Diseases” OR “Neurodegenerative Disorder” OR “Neurologic Degenerative Disease” OR “Neurologic Degenerative Condition” OR “Neurologic Degenerative Diseases” OR “Degenerative Neurologic Disease” OR “Alzheimer Disease” OR “Alzheimer Syndrome” OR “Alzheimer Type Dementia” OR “Senile Dementia” OR “Parkinson Disease” OR “Huntington Disease” OR “Chronic Progressive Hereditary Chorea” OR “Huntington Chorea” OR “Amyotrophic Lateral Sclerosis” OR “Frontotemporal Dementia” OR “Frontotemporal Lobe Dementia” OR “Multiple System Atrophy” OR “Spinocerebellar Ataxia” OR “Prion Diseases”). The gene editing and stem cell group integrated technical keywords as follows: TS= (“Gene Editing” OR “Genome Editing” OR “Base Editing” OR “Genetic Therapy” OR “Genetic Therapies” OR “DNA Therapy” OR “Gene Therapy” OR “Gene-Based Therapy” OR “Stem Cell Therapy” OR “Stem Cell Treatment” OR “Stem Cell Transplantation”). Moreover, the search terms made full use of the truncation symbol “*" to cover term variants (such as “therapy” and “therapies”). The literature screening was carried out in 2 stages: preliminary screening and manual screening. In the preliminary screening stage, nonresearch papers/review literature (such as conference abstracts and patents) and non-English literature were first excluded. In the manual screening stage, 2 researchers independently evaluated the literature, and they mainly excluded the literature that did not match the disease type and the degenerative lesions (such as stroke and epilepsy) and the literature that did not match the treatment methods (such as literature only involving traditional drugs or nongenetic intervention methods). When there were disagreements during the screening process, a consensus was reached through discussion to ensure that the finally included literature met both the relevance of the target disease and the consistency of the treatment methods. Finally, we exported the relevant data of the literature (authors, institutions, keywords, references, etc) in plain text format.

### Research Methods

We built a reproducible, multistep workflow and applied complementary software tools for descriptive statistics, collaboration mapping, and knowledge-structure mining. We first used HistCite (v12.03.17; developed by Eugene Garfield) to summarize annual publication output and overall growth of the field. To assess citation impact, we calculated the total local citation score (TLCS) and total global citation score (TGCS). The TLCS captures a paper’s influence within the dataset, whereas the TGCS reflects its impact across the broader scientific literature. Together, these metrics provide a domain-specific and field-wide view of research influence.

Next, we used VOSviewer (v1.6.19; developed by Nees Jan van Eck and Ludo Waltman at Leiden University’s Centre for Science and Technology Studies) to construct co-authorship networks at the country, institution, and author levels. To focus on core contributors, we applied minimum thresholds of 20 publications for countries and institutions and 10 publications for authors. In the resulting maps, node size is proportional to publication output, and edge thickness represents collaboration strength. Overlay visualizations encode the average publication year as a color gradient, allowing temporal shifts in activity to be viewed directly [18,19].

To characterize the intellectual structure and identify emerging fronts, we applied CiteSpace (v6.3.R1; developed by Chaomei Chen at Drexel University). We performed document co-citation clustering using 1-year time slices, selected nodes with the g-index (k=25), and pruned networks with the Pathfinder algorithm to emphasize the strongest connections. Cluster quality was evaluated using modularity (Q) and mean silhouette (S) values, with Q >0.3 indicating a meaningful modular structure and S >0.5 indicating acceptable internal consistency. All clustering analyses in this study met these criteria. We then used keyword co-occurrence and burst detection to define major themes and track rapidly rising topics. Finally, journal dual-map overlays were used to visualize cross-disciplinary citation flows [20].

## Results

### Annual Publication Trend Analysis

**Figure 1.**
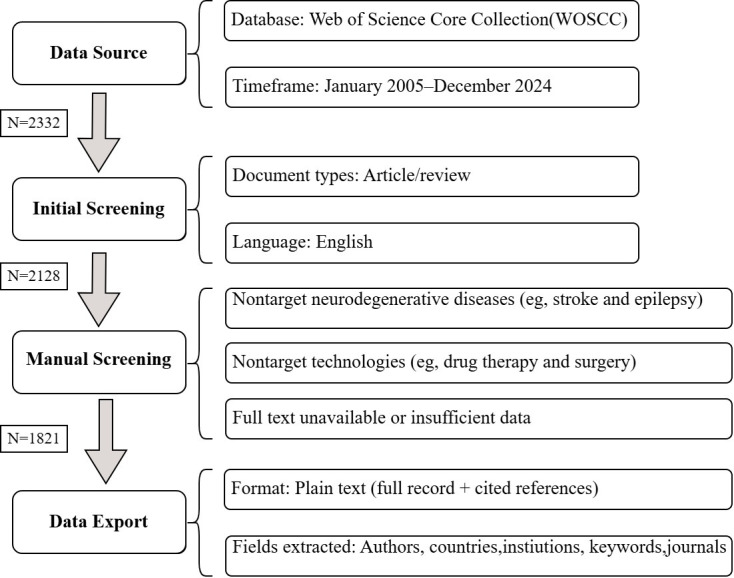
Flowchart of data collection in this study. The diagram illustrates the identification, screening, eligibility, and inclusion phases for records retrieved from the Web of Science Core Collection. The numbers of records identified, included, and excluded at each stage are shown, with specific reasons for exclusion provided in the corresponding boxes.

We identified 1821 publications (2005‐2024) (Figure 1), including 1083 original articles (59.5%) and 738 reviews (40.5%). Articles accumulated a TLCS of 3343 and a TGCS of 45,163, whereas reviews had a TLCS of 1443 and a TGCS of 34,729. The predominance of original articles is consistent with a technology-driven surge in primary discovery, while the substantial share of reviews suggests that multiple subareas have begun to consolidate into more mature themes. Citation patterns differed by publication type. Articles showed a lower TGCS-to-TLCS ratio than reviews (13.5 vs 24.1), indicating that original studies tended to concentrate influence within the core literature of this domain, while reviews more readily propagated across adjacent fields. Together, these data suggest a field still fueled by primary innovation but increasingly supported by integrative synthesis.

**Figure 2.**
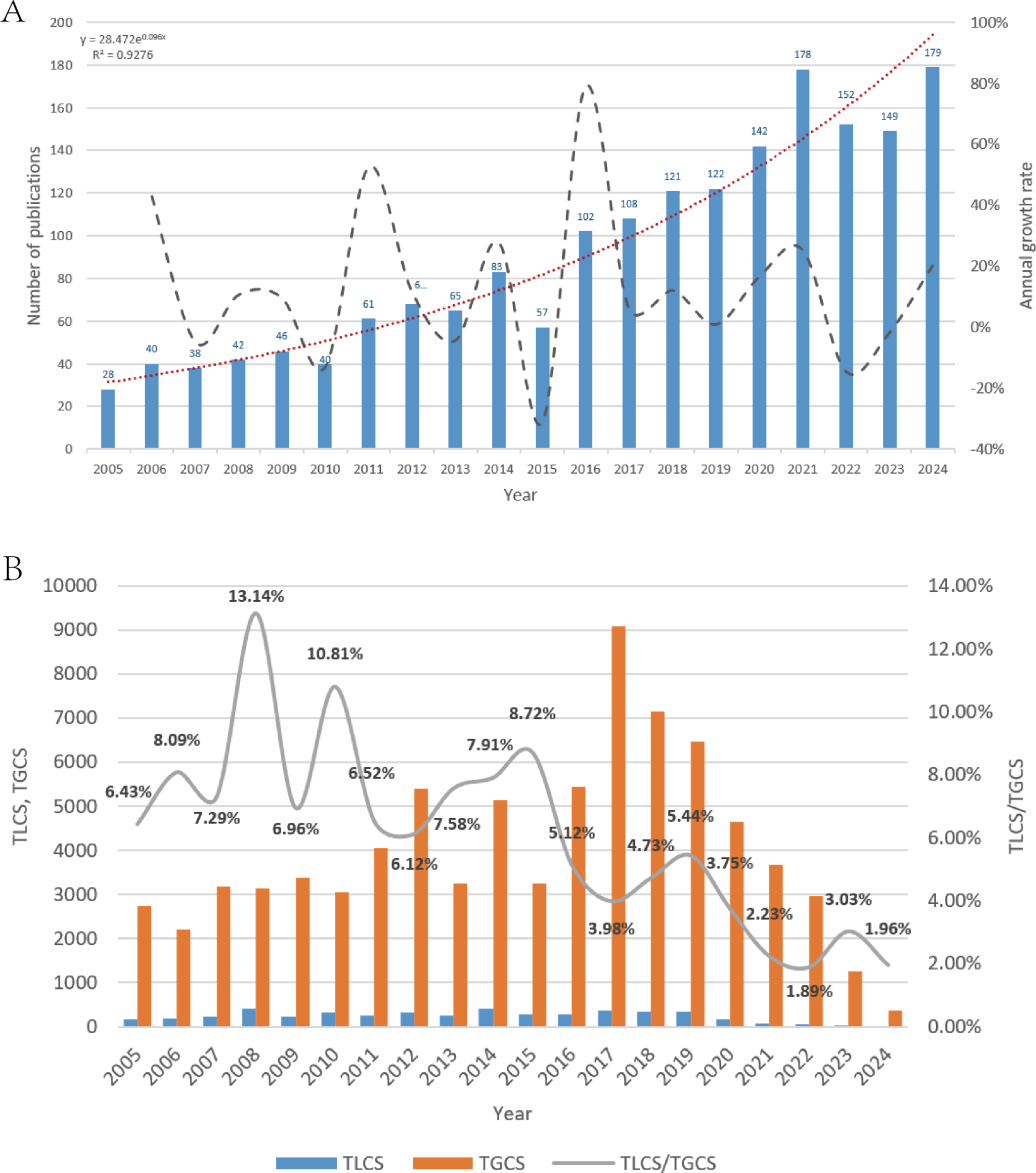
Annual publication trend. (A) Annual publication output, trend line, and growth rate from 2005 to 2024. Line chart showing the number of publications per year (bars, left y-axis) and the calculated annual growth rate percentage (line, right y-axis). The dashed trend line is fitted to the publication count. (B) Temporal distribution of the total local citation score (TLCS), total global citation score (TGCS), and TLCS/TGCS ratio. Blue bars represent the annual TLCS (left y-axis), orange bars represent the annual TGCS (left y-axis), and the line represents the TLCS/TGCS ratio (right y-axis).

Figure 2A summarizes the annual publication output, fitted trend, and growth rate from 2005 to 2024. Output increased from 28 publications in 2005 to 179 in 2024, with a stepwise rise over time. This trajectory is separated into 2 phases: an exploratory period (2005‐2015) with a mean of 57 publications per year, followed by an acceleration period (2016‐2024) with a mean of 125 publications per year. Figure 2B shows the annual TLCS, annual TGCS, and TLCS/TGCS ratio over the same time window. The annual TLCS peaked in 2012 (TLCS=413), coinciding with the highest TLCS/TGCS ratio (13.14%). In contrast, the annual TGCS reached its maximum value in 2017 (TGCS=9092), reflecting a period in which publications from this field achieved particularly broad citation uptake.

### Country Analysis

A total of 90 countries published work on gene editing and/or stem cell therapies for NDs. Table 1 summarizes the 10 most productive countries, including the publication count, share, TLCS, TGCS, and average number of citations per article. The United States ranked first by output (n=780) and by citation impact (TLCS=2784; TGCS=40,009), accounting for more than 40% of all publications in the dataset. Germany had the highest average number of citations per article (97.90). China and India ranked among the top 5 in publication volume but had the lowest average number of citations per article among leading countries, indicating high throughput but comparatively limited per-paper impact.

**Table 1.** Top 10 countries with the most publications.

Rank	Country	Documents (N=1821), n (%)	TLCS^a^	TGCS^b^	Average article citation
1	United States	780 (42.8)	2784	40,009	51.29
2	China	282 (15.5)	384	8180	29.01
3	Italy	146 (8.0)	448	6807	46.62
4	United Kingdom	138 (7.6)	262	9980	72.32
5	India	110 (6.0)	70	2159	19.63
6	France	91 (5.0)	214	4684	51.47
7	Japan	88 (4.8)	146	2462	27.98
8	Germany	80 (4.4)	102	7832	97.90
9	South Korea	79 (4.3)	257	2890	36.58
10	Canada	77 (4.2)	159	6690	86.88

a TLCS: total local citation score.

b TGCS: total global citation score.

**Figure 3.**
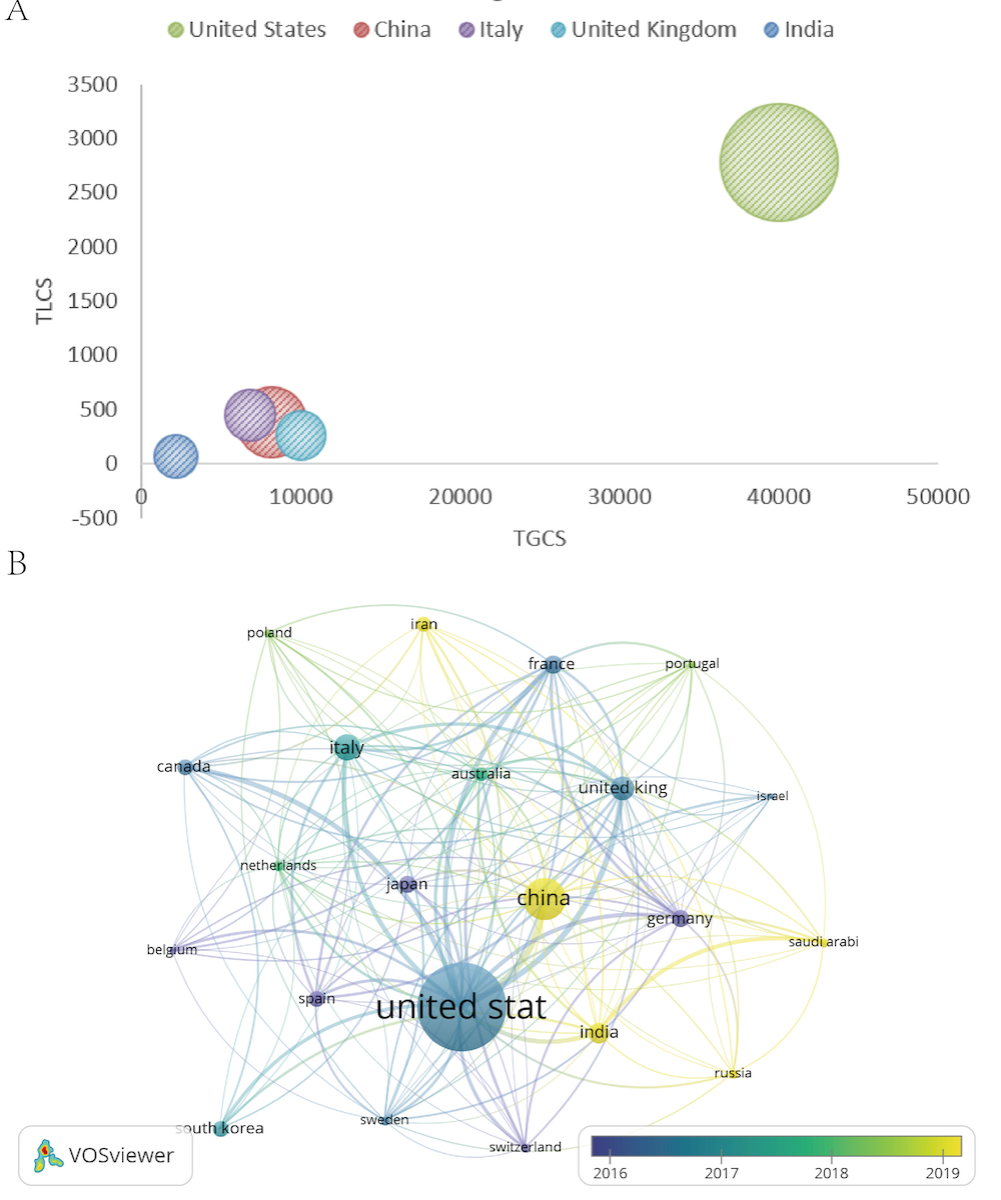
Country analysis. (A) Bubble chart of the top 5 countries by publication volume. The x-axis represents the total global citation score (TGCS) as a measure of cross-disciplinary reach, while the y-axis represents the total local citation score (TLCS), indicating field-specific recognition. The bubble size corresponds to the total publication count. (B) Country overlay visualization diagram. Nodes represent countries, with size proportional to the publication volume. Links indicate collaboration between countries. Node color denotes the average publication year.

Figure 3A visualizes the top 5 countries using a bubble plot in which the TGCS (cross-field reach) is shown on the x-axis and the TLCS (within-field influence) is shown on the y-axis; bubble size reflects publication volume. The United States clustered in the upper-right quadrant, consistent with strong performance across productivity and both citation dimensions. China ranked second by output but remained well below the United States in the TLCS and TGCS, suggesting that publication volume has outpaced citation influence. Italy and the United Kingdom produced similar numbers of papers but showed distinct citation profiles: the United Kingdom had a higher TGCS (9980 vs 6807), whereas Italy had a higher TLCS (448 vs 262), consistent with differences in how research from each country disseminates within versus beyond the core field. India showed a lower TLCS and TGCS overall, which may reflect differences in infrastructure, funding, and international visibility.

Country collaboration patterns are shown in the overlay visualization (Figure 3B). Nodes denote countries, edges indicate collaboration strength, node size represents publication volume, and node color reflects average publication year. The network reveals broad international connectivity, with the United States serving as a central hub. Several late-emerging contributors, including China, India, Iran, Saudi Arabia, and Russia, showed more recent activity, suggesting a gradual diversification of global participation in this area.

### Analysis of Research Institutions

**Figure 4.**
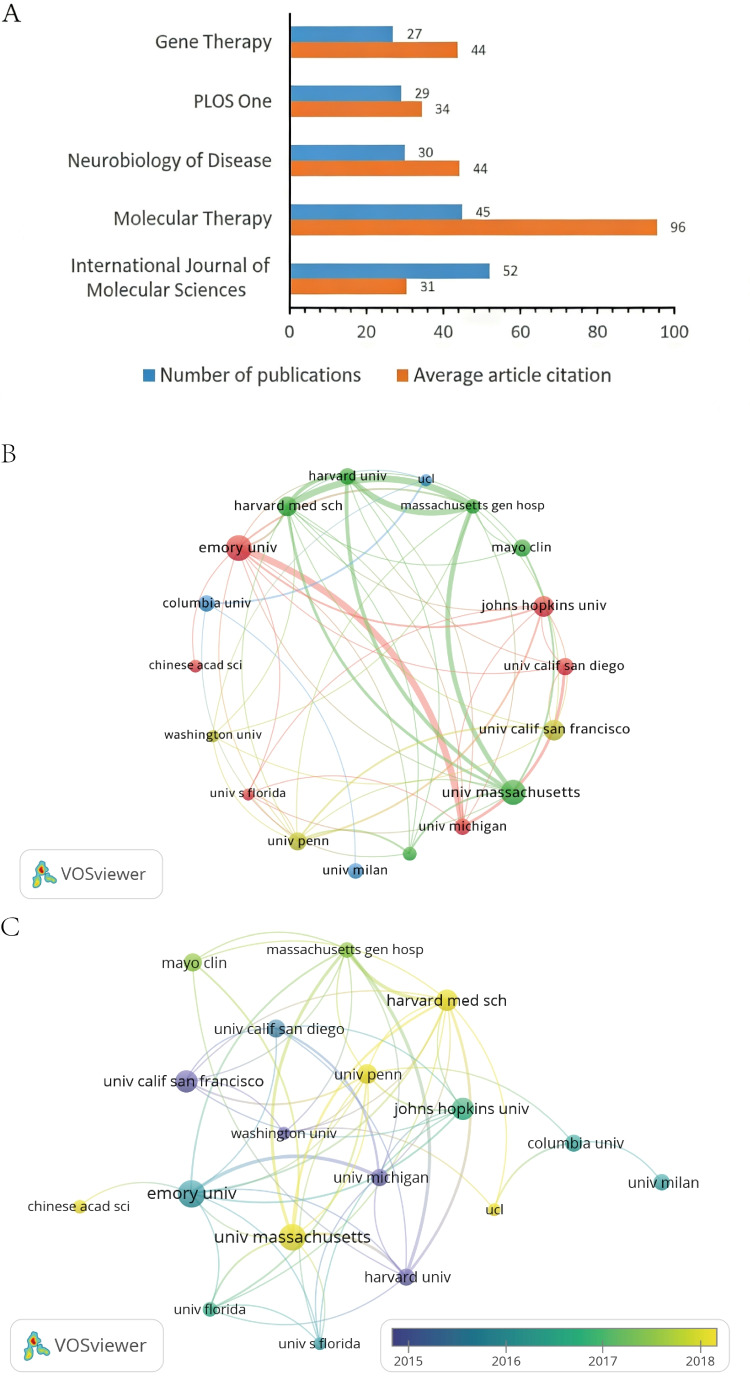
Research institution analysis. (A) The leading 5 institutions by publication volume and average citation impact. (B) Institutional collaboration network visualization diagram. Nodes represent institutions, with size proportional to the publication volume. Edges indicate co-authorship links, with thickness weighted by collaboration frequency. Only institutions with ≥20 publications are shown. (C) Institutional overlay visualization diagram. Overlay visualization map showing institutions colored by their average publication year, indicating shifts in research activity over time.

A total of 2515 institutions contributed to research on gene editing and/or stem cell therapies for NDs. The 5 most productive institutions are listed in Table 2; all these are based in the United States, consistent with the country-level dominance observed above. Publication counts across these top institutions were closely clustered (32‐41 papers), suggesting broad, parallel investment rather than concentration within a single “monopoly” center. Institution-level impact differed substantially. As shown in Figure 4A, the University of California, San Francisco, showed the highest average citations per paper (153.15), nearly threefold higher than Johns Hopkins University (55.73). This gap indicates that the University of California, San Francisco, not only had a high-volume output but also contributed disproportionately influential work within the field. Collaboration patterns are shown in Figure 4B. The national network highlights several strong institutional pairings, including Emory University-University of Michigan, Harvard University-University of California, San Francisco, and Massachusetts General Hospital–associated nodes. Together, these connections suggest an ecosystem in which basic research, technology development, and clinical programs are interlinked, supporting a “bench-to-clinic” pipeline. The overlay map (Figure 4C) further indicates a shift toward broader participation over time, with institutions, such as the University of Massachusetts, Harvard Medical School, the University of Pennsylvania, University College London, and the Chinese Academy of Sciences, appearing as increasingly active contributors in more recent years.

**Table 2.** Top 5 institutions with the most publications.

Rank	Institution	Country	Documents, n	Average article citation	TLCS^a^	TGCS^b^
1	Emory University	United States	41	49.34	310	2023
2	University of Massachusetts	United States	41	34.51	159	1415
3	Johns Hopkins University	United States	33	55.73	129	1839
4	University of California, San Francisco	United States	33	153.15	264	5054
5	Harvard Medical School	United States	32	32.31	69	1034

a TLCS: total local citation score.

b TGCS: total global citation score.

### Analysis of Research Authors

**Figure 5.**
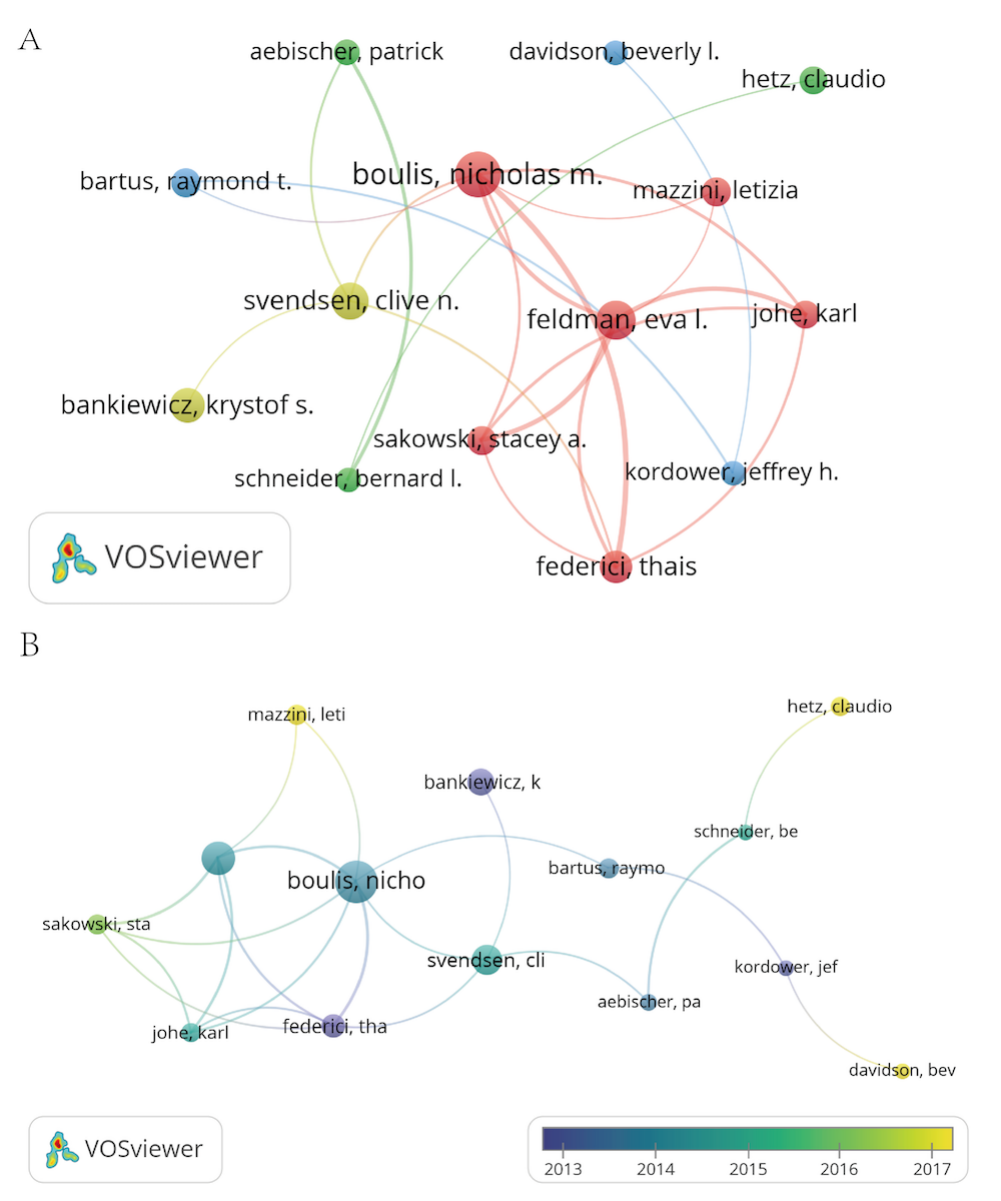
Research author analysis. (A) Author collaboration network visualization diagram. Nodes represent authors, sized by their publication count, and edges connect authors who have co-authored publications. Labels are shown for authors with ≥10 publications. (B) Author overlay visualization diagram. Overlay visualization of the author co-authorship network, with node color representing the average publication year of each author (gradient: blue=older, yellow=recent), highlighting authors active in different periods.

Across the 1821 publications, 9978 authors contributed to this research area. The 5 most productive authors are listed in Table 3. Boulis NM ranked first by publication count (24 papers) and contributed a highly cited clinical study in the *Annals of Neurology* (TGCS=157), “Intraspinal neural stem cell transplantation in ALS: phase 1 trial outcomes,” which established feasibility and safety for intraspinal neural stem cell delivery in ALS and helped shape subsequent translational efforts in the field [21]. Bankiewicz KS ranked second and showed the highest average number of citations per paper (124.07), consistent with sustained influence from work on aromatic L-amino acid decarboxylase (AADC) gene therapy for PD [22]. Feldman EL showed the highest H-index (104), reflecting broad and durable citation impact across multiple ND-related themes. Author collaboration is visualized in Figure 5A and B. Node sizes reflect publication volume, and the relatively small differences among the top authors point to a distributed authorship landscape rather than dominance by a single group. The overlay map further suggests increased activity around 2017, marked by the appearance of multiple highly active contributors (eg, Mazzini Leti, Davidson Bev, and Hetz Claudio), consistent with a period of accelerated field growth and diversification of research directions.

**Table 3.** Top 5 authors with the most publications.

Rank	Author	Documents, n	TLCS^a^	TGCS^b^	Average article citation	H-index	Organization
1	Boulis NM	24	223	1106	46.08	32	Emory University
2	Bankiewicz KS	14	267	1737	124.07	68	Ohio State University
3	Feldman EL	18	263	1064	59.11	104	University of Michigan
4	Svendsen CN	16	213	970	60.63	76	Cedars-Sinai Medical Center
5	Federici T	12	195	614	51.17	54	University of Milan

a TLCS: total local citation score.

b TGCS: total global citation score.

### Journal Analysis

The 1821 publications were distributed across 649 journals. The 5 most productive journals are summarized in Table 4. Collectively, these data illustrate a stratified dissemination landscape in which publication volume and citation influence do not always align. The *International Journal of Molecular Sciences* published the highest number of papers (n=52), but showed a modest impact factor (4.9) relative to other core journals, alongside 1586 total citations and an H-index of 114. This pattern suggests high throughput with more variable per-paper influence. In contrast, *Molecular Therapy* combined substantial output (45 papers) with the strongest citation profile among the leading journals (4300 total citations; impact factor of 12.1; H-index of 158), supporting its position as a central venue for high-impact work in this domain. *Neurobiology of Disease* ranked third (30 papers; impact factor of 5.1; 1327 citations; H-index of 151), consistent with sustained influence from mechanistic and disease-model studies. *PLOS One* ranked fourth (29 papers; impact factor of 2.9; 996 citations; H-index of 268), reflecting broad topical coverage and extensive long-term citation accumulation. *Gene Therapy* ranked fifth (27 papers; impact factor of 4.6; 1179 citations; H-index of 153), maintaining a stable niche within gene therapy research.

**Table 4.** Top 5 journals by publication volume.

Rank	Journal	Total publications, n	Total citations, n	Impact factor	H-index
1	*International Journal of Molecular Sciences*	52	1586	4.9	114
2	*Molecular Therapy*	45	4300	12.1	158
3	*Neurobiology of Disease*	30	1327	5.1	151
4	*PLOS One*	29	996	2.9	268
5	*Gene Therapy*	27	1179	4.6	153

**Figure 6.**
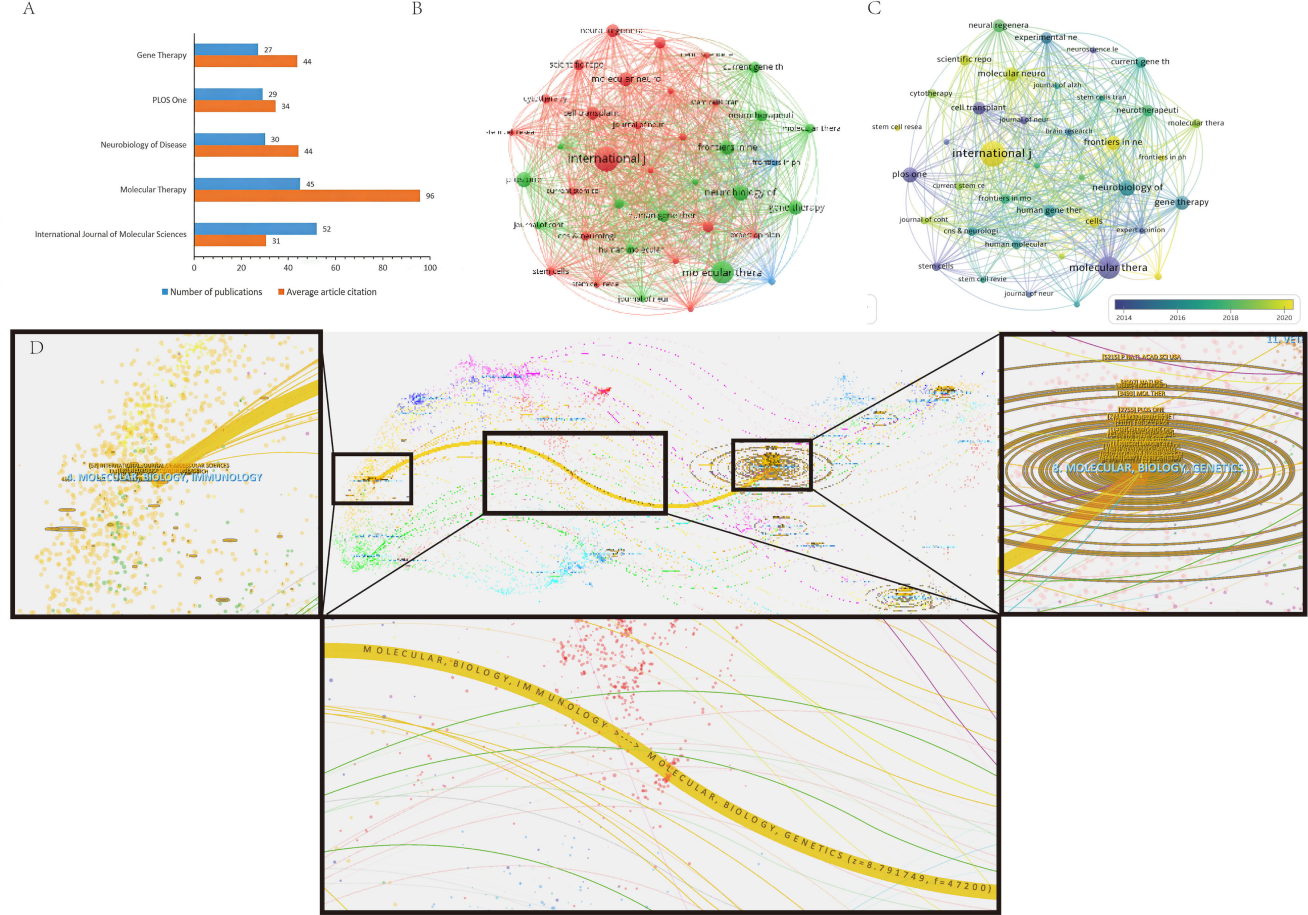
Journal analysis. (A) Top 5 journals by publication volume and field-weighted citation impact. Journal names are listed on the vertical axis. Blue bars represent the total number of publications per journal. Orange bars represent the average citations per article for that journal within the analyzed dataset. (B) Journal collaboration network visualization diagram. (C) Journal overlay visualization diagram. (D) Dual-map overlay of journals. Left-side clusters (citing journals) are concentrated in molecular biology/immunology and health/medicine, while right-side clusters (cited journals) are focused on molecular biology/genetics and clinical medicine. Colored curves indicate citation links, with curve thickness proportional to citation frequency (f). Key metrics: z=8.79, f=47,200.

Figure 6A compares publication volume and mean citations per paper for these journals and again highlights *Molecular Therapy* as a top contributor by both output and influence (45 papers; 95.56 citations per paper). Its most cited paper (226 citations), “Direct muscle delivery of glial cell line–derived neurotrophic factor (GDNF) via human mesenchymal stem cells (MSCs) improves motor neuron survival and function in a rat model of familial ALS,” reported the therapeutic benefit of GDNF-expressing human MSCs in an ALS rat model and serves as a representative translational milestone in this space [23]. Figure 6B shows dense interjournal citation connectivity, consistent with rapid knowledge turnover and active cross-referencing across core venues. The dual-map overlay (Figure 6C) indicates directional citation flows from citing journals focused on molecular biology and immunology toward citing journals focused on molecular biology and genetics, supporting a convergence of delivery/immune considerations with genetic and mechanistic foundations.

### Literature Co-Citation Analysis

To identify foundational studies shaping the field, we examined highly influential documents by the TLCS. Table 5 lists the 5 papers with the highest TLCS, which collectively span gene therapy approaches (including adeno-associated virus [AAV]–mediated glutamate decarboxylase [GAD]/AADC delivery) and stem cell–based strategies (including MSC transplantation). These studies also represent a range of administration routes (eg, intrathecal, intravenous, and intracerebral delivery) and translational stages from animal models to phase I clinical testing.

**Table 5.** Top 5 publications with the highest local total citation score.

Rank	Title	Journal	First author	Year	TLCS^a^
1	A phase 1 clinical trial of nerve growth factor gene therapy for Alzheimer disease [24]	*Nature Medicine*	Tuszynski MH	2005	102
2	Safety and tolerability of gene therapy with an adeno-associated virus (AAV) borne GAD gene for Parkinson’s disease: an open label, phase I trial [25]	*Lancet*	Kaplitt MG	2007	95
3	Mesenchymal stem cell transplantation in amyotrophic lateral sclerosis: a phase I clinical trial [26]	*Experimental Neurology*	Mazzini L	2010	86
4	Safety and immunological effects of mesenchymal stem cell transplantation in patients with multiple sclerosis and amyotrophic lateral sclerosis [27]	*Archives of Neurology*	Karussis D	2010	71
5	Safety and tolerability of putaminal AADC gene therapy for Parkinson disease [22]	*Neurology*	Christine CW	2009	65

a TLCS: total local citation score.

**Figure 7.**
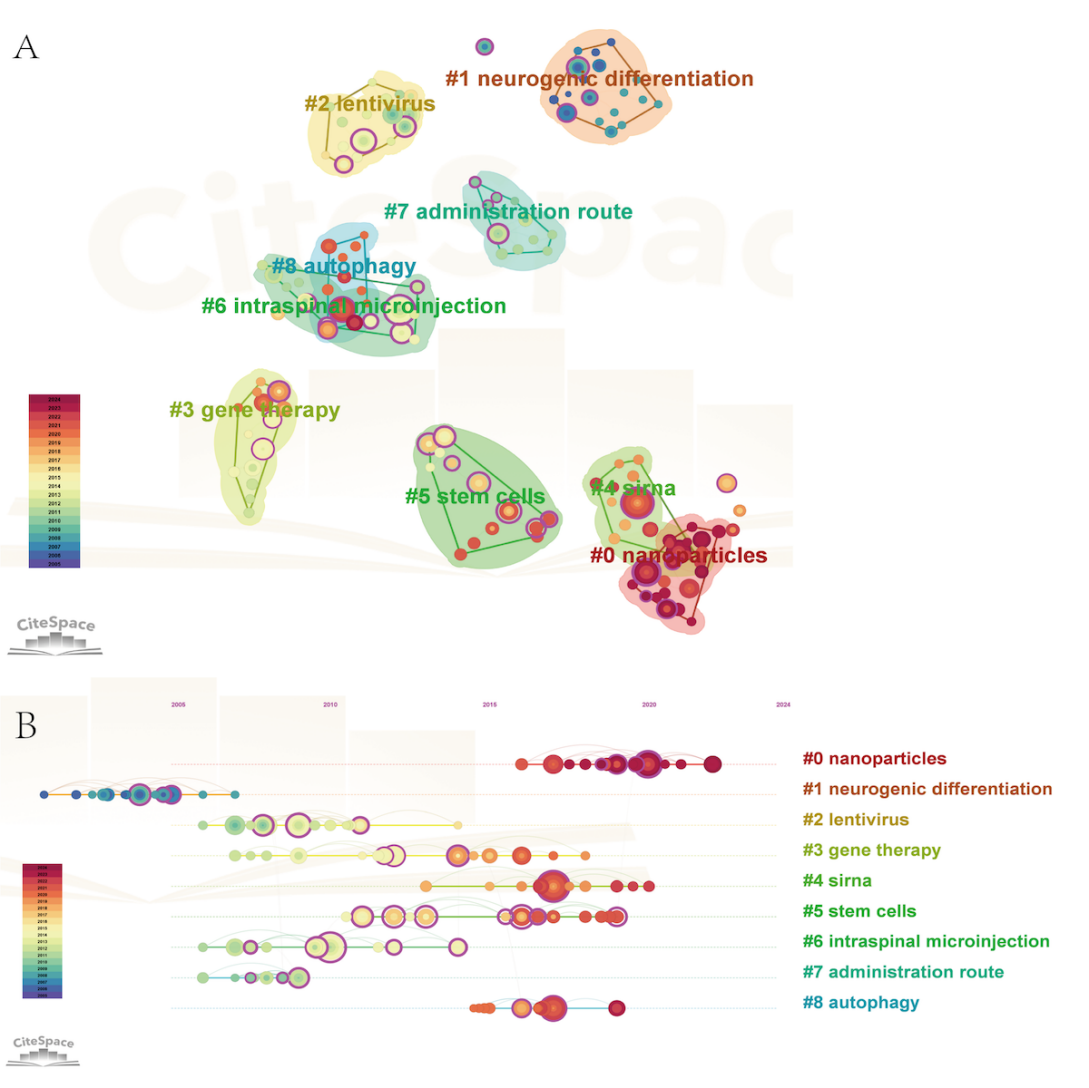
Co-citation analysis. (A) Clustering diagram of co-cited documents. Network of co-cited references clustered using CiteSpace. Nodes represent references, and links represent co-citation strength. Colors denote distinct thematic clusters (see cluster labels #0-#8). Clustering quality metrics: modularity Q=0.8485; mean silhouette S=0.9513. (B) Time evolution diagram of clustering of co-cited documents. Timeline visualization of the co-citation clusters shown in part A. The x-axis represents the publication year (2005-2024). Each horizontal band corresponds to a cluster, showing the timespan during which its constituent references were cited. Clusters are labeled #0-#8.

CiteSpace co-citation clustering revealed a strong network structure and cluster coherence (Q=0.8485; S=0.9513; Figure 7A). Nine major clusters were identified, including “nanoparticles,” “neurogenic differentiation,” “lentivirus,” “gene therapy,” “siRNA,” “stem cells,” “intraspinal microinjection,” “administration route,” and “autophagy,” collectively outlining the core intellectual pillars of the field. The timeline view (Figure 7B) further suggests that “nanoparticles,” “siRNA,” and “autophagy” have become more prominent in recent years, consistent with increasing emphasis on delivery engineering and mechanistic modulation.

### Keyword Analysis

**Figure 8.**
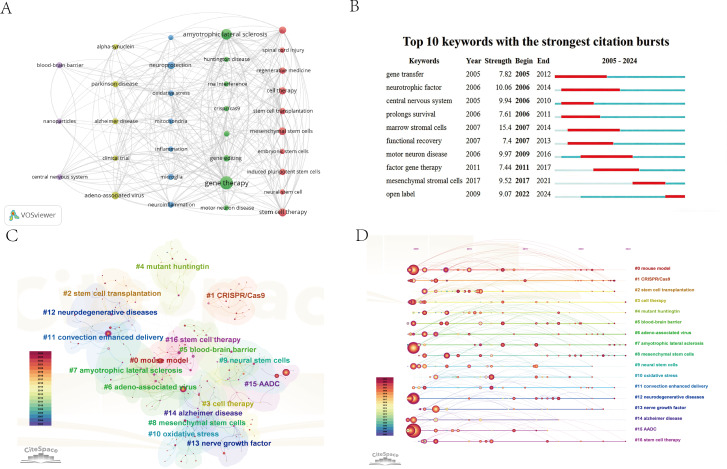
Keyword analysis. (A) Keyword co-occurrence network diagram. Node size corresponds to keyword frequency. Keywords are grouped by color based on co-occurrence patterns. (B) Keyword burst diagram. Burst detection analysis performed in CiteSpace (*γ*=0.5). Each horizontal bar represents a keyword, with red segments indicating the period of significant frequency increase (“burst”). (C) Keyword clustering diagram. Network of co-occurring keywords clustered using CiteSpace. Colors denote 17 distinct thematic clusters (labeled #0-#16). Clustering quality metrics: modularity Q=0.7813; mean silhouette S=0.9197. (D) Keyword clustering time evolution diagram. Timeline view of the keyword clusters from part C, showing the activity period of each cluster from 2005 to 2024. The x-axis represents time, and each horizontal line corresponds to a cluster (#0-#16), visualizing the emergence and persistence of research themes.

Keywords provide a compact representation of study focus and are well-suited for tracking thematic evolution. Figure 8A shows 31 keywords occurring more than 20 times, highlighting recurring attention to AAV, autophagy, blood-brain barrier (BBB), CRISPR/Cas9, iPSCs, and nanoparticles. Keyword co-occurrence analysis yielded high clustering quality (Q=0.7813; S=0.9197), indicating that the identified keyword groups are both well separated and internally consistent.

Burst detection (Figure 8B) identified “marrow stromal cells” as the strongest burst term (strength=15.4), while “neurotrophic factor” showed the longest burst duration (2006‐2014), consistent with an early wave of neuroprotection-focused strategies. Notably, “open label” emerged as a recent burst term, aligning with increasing attention to early-stage clinical translation.

Keyword clustering further resolved 17 clusters (Figure 8C), spanning disease entities and mechanisms (eg, NDs, ALS, AD, mutant huntingtin, and oxidative stress), gene editing and gene therapy platforms (eg, CRISPR/Cas9, AAV, and AADC), and stem cell or cell therapy strategies (eg, stem cell transplantation, MSCs, and neural stem cells). Additional clusters captured experimental models and neuroprotection-related themes (eg, mouse model and nerve growth factor) as well as delivery paradigms (eg, BBB and convection-enhanced delivery [CED]). The temporal map (Figure 8D) indicates sustained activity across multiple directions over the past 2 decades, with concurrent advances in delivery optimization, mechanistic deepening, and clinical translation. Together, these trends point to a field with not only strong foundational accumulation but also persistent bottlenecks that continue to motivate technological innovation and cross-disciplinary integration.

## Discussion

The treatment of NDs is at a critical inflection point, with gene editing and stem cell therapy increasingly converging as translational strategies. To our knowledge, this study is the first bibliometric analysis to systematically map the global research landscape and emerging trends in this field. Over the past 2 decades, annual publication output increased in a stepwise fashion and entered a clear acceleration phase after 2016. This shift likely reflects several enabling advances in gene and cell therapy. For example, the rAAV2-retro variant, which has been generated through AAV capsid engineering, can efficiently access projection neurons via retrograde transport, improving gene delivery performance [28]. In parallel, iPSCs became essential for disease modeling, and MSCs engineered to overexpress brain-derived neurotrophic factor (BDNF) improved outcomes in HD mouse models [29]. Together, these developments likely contributed to sustained growth from 2016 onward. High-impact outputs in this area also show recognizable milestone patterns. The local citation peak in 2008 (TLCS=413; TLCS/TGCS=13.14%) may relate to studies reporting that MSC transplantation can prolong survival, reduce neuroinflammation, and improve motor neuron function in ALS mouse models [30,31]. In contrast, the global citation peak in 2017 (TGCS=9092) likely reflects broad uptake of the widely cited review “Parkinson disease” in *Nature Reviews Disease Primers* (2017), which synthesized gene therapy targets and vectors as well as the trajectory and current status of stem cell–based strategies in PD [32]. These peaks appear to align with 2 key stages of field development: proof-of-concept experimental validation and consolidation of translational pathways.

At the country level, the United States maintains a dominant position in publication output as well as in the TLCS and TGCS, and the top 5 institutions are all based in the United States. The Boston area, anchored by Harvard University and Massachusetts General Hospital, exemplifies a “basic-to-clinical” collaborative ecosystem, where geographic proximity may facilitate high-frequency interaction and accelerate translation. The sustained US advantage likely reflects multiple reinforcing factors. Long-term and stable research and development investment provides resilience for high-risk, long-cycle research and supports continuity in key directions. In addition, policy and institutional infrastructure (eg, federal funding mechanisms, evaluation systems, open science practices, and industry-academia translation pathways) may improve efficiency across the pipeline—from project initiation to dissemination—thereby strengthening both output and impact. Finally, the centrality of US institutions within international collaboration networks increases access to high-impact consortia and may amplify visibility and downstream citation performance.

China and India contribute substantial publication volume, but their per-paper citation impact remains comparatively low (China: 248 articles; 29.01 citations per article; India: 110 articles; 19.63 citations per article). Collaboration structure may partly explain this gap. China’s collaboration intensity with Western countries remains far below that of the United States (Figure 3B), and India appears more peripheral in the international network, potentially limiting exchange and dissemination. Journal distribution may also contribute. As shown in Table 4 and Figure 6A, outputs from these countries more often appear in high-volume journals with lower field-specific influence, whereas representation in specialty high-impact venues, such as *Molecular Therapy*, remains limited. In addition, keyword evolution (Figures 7 and 8) suggests that research from these countries may concentrate more heavily on applied and translational clusters, while fewer outputs appear to define foundational, concept-setting advances that often drive long-term citation accumulation. Collectively, these patterns suggest that increasing influence will require deeper integration into high-impact international networks and more sustained investment in originality and frontier research.

At the same time, our data suggest a complementary global innovation landscape. The United States shows broad leadership with strong representation in foundational and early-translational clusters, such as novel AAV engineering and first-in-human studies, consistent with its network position and presence in high-impact journals [33]. In contrast, publication growth from countries, such as China and India, aligns more strongly with applied technology clusters, including nanoparticle delivery systems and stem cell transplantation protocols [34]. European contributors, including Germany and the United Kingdom, show sustained depth in mechanistic clusters, such as oxidative stress and protein aggregation, reflecting enduring strengths in fundamental biomedical science [35]. These patterns argue for collaboration models that pair mechanistic depth, platform engineering, and clinical execution. Institutions seeking higher impact may benefit from long-term partnerships with leading laboratories through co-designed projects and joint publication strategies, alongside deliberate targeting of field-leading specialty journals and sustained investment in frontier directions.

Our co-citation and keyword analyses highlight recurring focal areas, including “adeno-associated virus,” “nanoparticles,” “blood-brain barrier,” and “marrow stromal cells.” AAV vectors remain central tools due to relatively low immunogenicity, broad tropism, and stable long-term transgene expression [36]. We also identified clusters, such as “lentivirus” and “siRNA.” Lentiviral vectors can enable durable gene expression through genomic integration and provide relatively large cargo capacity for platforms such as CRISPR/Cas9 or neurotrophic factor genes [37,38]. Small interfering RNA (siRNA) can silence pathogenic genes through RNA interference and may also complement gene-editing strategies by modulating target expression or reducing unintended activity [39]. However, clinical translation remains constrained by 2 major bottlenecks [40]. First, BBB penetration is often insufficient in humans, despite robust performance in some animal studies. Second, neutralizing antibodies against AAV can reduce transduction efficiency and may contribute to treatment failure, while immune-evasion strategies remain comparatively underdeveloped. Recent progress in surface-modified and targeted nanoparticles has improved BBB delivery [41]. In our analyses, “nanoparticles” emerged as both a persistent focus and a growing frontier. Nanoparticles (1‐100 nm) can be engineered (eg, using rabies virus glycoprotein peptides or lactoferrin) to facilitate BBB transport; protect payloads, such as AAV and siRNA; and potentially influence stem cell survival and differentiation [42,43]. Encapsulating AAV within lipid nanoparticles, for example, has been reported to enhance vector stability and in vivo penetration through optimization of lipid composition and structure [44]. These advances underscore the promise of integrating material science with vector biology to improve delivery and the therapeutic index.

Another prominent cluster was “autophagy,” a cellular quality-control pathway that clears misfolded proteins and damaged organelles. Enhancing autophagy is an attractive therapeutic direction, but precise bidirectional control remains challenging because excessive activation risks degrading essential proteins [45]. MSCs and MSC-derived extracellular vesicles also remain highly visible in the literature. The burst term “marrow stromal cells” supports sustained attention to MSC-based strategies. Bone marrow–derived MSCs can support neural repair through the secretion of neurotrophic factors and immunomodulatory effects [46]. Human MSCs engineered to overexpress BDNF improved outcomes in HD mouse models [29,47]. Laromestrocel, an allogeneic bone marrow–derived MSC product from young healthy donors, has shown encouraging findings in AD, including reduced rates of whole-brain volume loss and hippocampal atrophy, without amyloid-related imaging abnormalities during treatment [48]. As a cell-free alternative, MSC-derived exosomes are emerging as nanoscale carriers capable of delivering therapeutics to the brain, supporting Aβ clearance and neuroprotection [49]. MSC-derived exosomes have also been reported to localize to inflamed brain regions and to be taken up by neurons, suggesting potential for targeted delivery [50]. The recent emergence of “open label” indicates that clinical translation is advancing but still concentrated in early validation. Consistent with this, many highly cited clinical studies in this field are phase I open-label trials. While open-label designs are practical for early exploration, definitive efficacy requires subsequent confirmation in larger randomized, double-blind controlled studies [24-27]. Beyond vector-related barriers, translation also faces platform-wide challenges: nanoparticle safety and long-term stability in humans remain incompletely defined; MSC survival and integration after transplantation are often limited; gene editing carries the risk of off-target effects; and stem cell–based approaches raise concerns regarding tumorigenicity and immune reactions. Finally, many trials remain limited by a small sample size and a short follow-up, underscoring the need for larger cohorts and longer-term surveillance to establish durability and rare safety signals.

Our keyword clustering further resolved thematic dimensions beyond tools and cells. These included disease entities and mechanisms (eg, oxidative stress, mutant huntingtin, AD, and ALS), gene-editing and gene-therapy platforms (CRISPR/Cas9, AAV, and AADC), stem cell and cell therapy strategies (MSCs, neural stem cells, and transplantation), delivery paradigms (CED and BBB), and experimental models and neuroprotection-related themes (mouse models and nerve growth factor). Oxidative stress reflects cellular injury driven by excessive reactive oxygen and nitrogen species, and interventions that neutralize reactive oxygen species remain under active exploration, although deeper mechanistic work is still needed to guide therapeutic development. CRISPR/Cas9 enables precise genome manipulation [51], and AADC gene therapy has shown translational potential in PD, although dose optimization and immunogenicity remain important challenges. Stem cell therapy offers multitarget potential but continues to face constraints in standardized manufacturing, delivery efficiency, and long-term safety [52-56]. Delivery remains a central determinant of success: CED can improve distribution within the central nervous system and offers a strategy to address BBB-associated constraints [57,58]. Animal models remain essential, but differences between rodents and humans, particularly BBB properties and lifespan, can contribute to translational inconsistency. Clinical studies of neurotrophic factors, such as nerve growth factor, have generally shown safety but limited efficacy in late-stage disease, highlighting the complexity of these interventions [59]. Overall, progress will depend on improving delivery and efficiency, reducing immune barriers, and strengthening safety assessments to support reliable clinical benefit.

The integration of iPSC technology with CRISPR/Cas9 editing remains particularly promising for personalized approaches. Patient-derived iPSCs can mitigate immune rejection concerns while enabling gene correction or targeted modification. In HD, CRISPR/Cas9 correction of CAG-repeat expansions in *HTT* in patient-derived iPSCs yielded differentiated neurons with improved functional phenotypes [60]. Additionally, iPSCs can differentiate into diverse neural cell types, and when combined with CRISPR/Cas9 technology, they enable the generation of specialized cells tailored for multicell-type ND therapies. In PD treatment, researchers differentiated iPSCs into dopaminergic neurons, applied CRISPR-mediated gene correction, and observed symptom improvement in PD models following transplantation [61]. In AD, neurons or astrocytes can be obtained through differentiation of patient-derived iPSCs, and CRISPR/Cas9 can be used for precise knock-in/out or allele replacement of key risk genes, such as *APP*, *PSEN1/2*, and *APOE*, thereby reversing most phenotypes [62]. In ALS, pathogenic variants around *SOD1*, *C9ORF72*, *TARDBP*, and *FUS* can be investigated in motor neurons differentiated from iPSCs and their co-culture systems, observing key phenotypes such as RNA metabolism abnormalities, stress granules, axonal transport deficits, and cell nonautonomous damage, as well as using gene correction to validate the causal relationship between phenotypes and mutations [63]. Across multiple ND models, combined iPSC-CRISPR strategies have shown therapeutic benefit with acceptable tolerability and low immunogenicity in animal studies [64].

In the process of transitioning technologies from the laboratory to clinical and industrial applications, ethical considerations and regulatory frameworks often pose more immediate practical bottlenecks, impacting whether new technologies can gain approval, establish trust, and achieve large-scale implementation, in addition to scientific feasibility and engineering maturity. Ethically, it is essential to regulate informed consent, privacy, and data security while ensuring fairness in the risk-benefit balance. In the context of iPSCs and CRISPR, particular attention should be given to evaluating off-target or unintended effects, long-term safety, follow-up requirements, and the protection of vulnerable populations. On the regulatory front, this involves product attributes, quality systems, and the requirements for clinical evidence and risk management, including endpoint settings and long-term safety evaluations. These factors significantly influence research design, data availability, timelines, and costs, ultimately determining the efficiency of translation.

This study has several limitations. First, literature retrieval was restricted to the WoSCC, which may omit journals or regional contributions not indexed in this database. Second, although search terms were refined, omissions cannot be fully excluded. Third, we did not incorporate additional translational indicators, such as patent landscapes.

This study systematically reveals dynamic development trends and research hotspots in gene editing and stem cell therapy for NDs through bibliometric analysis. Global annual publications show stepwise growth, with countries like the United States and Germany making outstanding contributions. However, clinical translation faces challenges, such as low BBB penetration efficiency and immune responses, though emerging approaches like nanoparticle-rabies virus glycoprotein peptide modification and MSC exosomes demonstrate breakthrough potential. Additionally, combined applications of iPSCs and CRISPR/Cas9 gene editing show significant therapeutic promise. The “open label” keyword burst indicates that most studies remain in early validation phases, characterized by small clinical trial samples, short observation periods, and weak evidence levels, necessitating urgent validation of long-term safety and efficacy. Future efforts should prioritize optimizing delivery systems, validating clinical translation, and fostering interdisciplinary research (eg, artificial intelligence–driven AAV capsid design) through dedicated ND funds. Strengthening “basic-clinical-industrial” collaborative networks, enhancing clinical evidence quality, and exploring CRISPR/Cas9-stem cell integration will provide critical pathways to overcoming therapeutic bottlenecks in NDs.

## Abbreviations

AADC: aromatic L-amino acid decarboxylase
AAV: adeno-associated virus
AD: Alzheimer disease
ALS: amyotrophic lateral sclerosis
Aβ: amyloid-β
BBB: blood-brain barrier
BDNF: brain-derived neurotrophic factor
Cas9: CRISPR-associated protein 9
CED: convection-enhanced delivery
CRISPR: clustered regularly interspaced short palindromic repeats
GDNF: glial cell line–derived neurotrophic factor
HD: Huntington disease
iPSC: induced pluripotent stem cell
MSC: mesenchymal stem cell
ND: neurodegenerative disease
PD: Parkinson disease
siRNA: small interfering RNA
TGCS: total global citation score
TLCS: total local citation score
WoSCC: Web of Science Core Collection
